# Strong B-T inverse

**DOI:** 10.1016/j.heliyon.2024.e38580

**Published:** 2024-09-30

**Authors:** Sanzhang Xu, Yuyue Huang, Jinyong Wu, Ber-Lin Yu

**Affiliations:** a Faculty of Mathematics and Physics, Huaiyin Institute of Technology, Huaian 223003, China; b School of Mathematics and Physics, Wenzhou University, Wenzhou 325035, China

**Keywords:** 15A09, Generalized inverse, B-T inverse, Hartwig-Spindelböck decomposition

## Abstract

New-typed matrix inverses based on the Hartwig-Spindelböck decomposition were investigated, which is called the strong B-T inverse, as a generalization of the B-T inverse. The relationships between the above inverse and other matrix inverses were established. Several sufficient and necessary conditions of the strong B-T inverse are obtained via the column and null spaces.

## 1 Introduction

The symbol $C^{m\times n}$ is the set of all m×n complex matrices over the filed C. Notations $A^{⁎}={\bar{A}}^{⊤}$, R(A) is the column space of *A* and N(A) is the null space of *A*. The number *k* is called the index of *A* (denoted by ind(A)) if *k* is the smallest positive integer such that $\mathrm{rank}(A^{k})=\mathrm{rank}(A^{k+1})$. The symbol $A^{†}$ denotes the Moore-Penrose inverse of *A* [8], [10].

Every matrix $A\in C^{n\times n}$ with rank(A)=r has the following form

(1.1) $$A=U\left[\begin{array}{cc} \Sigma K\; & \Sigma L\\ 0\; & 0 \end{array}\right]U^{⁎},$$

where $UU^{⁎}=E_{n}$, $\Sigma =\sigma_{1}E_{r_{1}}⊕\cdots ⊕\sigma_{t}E_{r_{t}}$ is a diagonal matrix, where $\sigma_{i}(i=1,2,\cdots ,r)$ are nonzero singular values of *A* and $\sigma_{1}>\sigma_{1}>\cdots >\sigma_{t}>0$, $r_{1}+\cdots +r_{t}=r$. Moreover, we have $KK^{⁎}+LL^{⁎}=E_{r}$, where $K\in C^{r\times r}$ and $L\in C^{r\times (n-r)}$. This decomposition in (1.1) is called the Hartwig-Spindelböck decomposition (or called the Σ-K-L decomposition) [5].

In [2, Definition 1], the definition of the B-T inverse of *A* was given, which is related ${\left(A^{2}A^{†}\right)}^{†}$ and denoted by $A^{⋄}$. In [2, Corollary], the equality $A^{⋄}AA^{⋄}=A^{⋄}$ was proved.

## 2 Preliminaries relate to the Σ-K-L decomposition

Lemma 2.1 [1, p2799 (1.4)] *If* $A\in C^{n\times n}$ *has the decomposition as given in* (1.1) *, then the expression of the Moore-Penrose inverse is*

(2.1) $$A^{†}=U\left[\begin{array}{cc} K^{⁎}\Sigma^{-1}\; & 0\\ L^{⁎}\Sigma^{-1}\; & 0 \end{array}\right]U^{⁎}.$$

Lemma 2.2 [2, Lemma 2] *If* $A\in C^{n\times n}$ *has the decomposition as given in* (1.1) *, then the expression of the B-T inverse is*

$$A^{⋄}=U\left[\begin{array}{cc} {\left(\Sigma K\right)}^{†}\; & 0\\ 0\; & 0 \end{array}\right]U^{⁎}.$$

## 3 Strong B-T inverse

Lemma 3.1 *Let* $A\in C^{n\times n}$ *and* $\alpha_{A}=AA^{†}+{\left(A^{†}\right)}^{⁎}(E_{n}-AA^{†})$ *. If A has the Hartwig-Spindelböck decomposition as given in* (1.1) *, then*

$$\alpha_{A}=U\left[\begin{array}{cc} E_{r}\; & \Sigma^{-1}L\\ 0\; & 0 \end{array}\right]U^{⁎},$$

*where* $E_{r}$ *is the identity matrix of size r.*

Proof By equality (2.1), we have

$$\alpha_{A}=AA^{†}+{\left(A^{†}\right)}^{⁎}(E-AA^{†})=U\left[\begin{array}{cc} \Sigma K\; & \Sigma L\\ 0\; & 0 \end{array}\right]\left[\begin{array}{cc} K^{⁎}\Sigma^{-1}\; & 0\\ L^{⁎}\Sigma^{-1}\; & 0 \end{array}\right]U^{⁎}+U{\left[\begin{array}{cc} K^{⁎}\Sigma^{-1}\; & 0\\ L^{⁎}\Sigma^{-1}\; & 0 \end{array}\right]}^{⁎}\left(\left[\begin{array}{cc} E_{r}\; & 0\\ 0\; & E_{n-r} \end{array}\right]-\left[\begin{array}{cc} \Sigma K\; & \Sigma L\\ 0\; & 0 \end{array}\right]\left[\begin{array}{cc} K^{⁎}\Sigma^{-1}\; & 0\\ L^{⁎}\Sigma^{-1}\; & 0 \end{array}\right]\right)U^{⁎}=U\left[\begin{array}{cc} KK^{⁎}+LL^{⁎}\; & 0\\ 0\; & 0 \end{array}\right]U^{⁎}+U\left[\begin{array}{cc} \Sigma^{-1}K\; & \Sigma^{-1}L\\ 0\; & 0 \end{array}\right]\left(\left[\begin{array}{cc} E_{r}\; & 0\\ 0\; & E_{n-r} \end{array}\right]-\left[\begin{array}{cc} \Sigma (KK^{⁎}+LL^{⁎})\Sigma^{-1}\; & 0\\ 0\; & 0 \end{array}\right]\right)U^{⁎}=U\left[\begin{array}{cc} E_{r}\; & 0\\ 0\; & 0 \end{array}\right]U^{⁎}+U\left[\begin{array}{cc} \Sigma^{-1}K\; & \Sigma^{-1}L\\ 0\; & 0 \end{array}\right]\left[\begin{array}{cc} 0\; & 0\\ 0\; & E_{n-r} \end{array}\right]U^{⁎}=U\left[\begin{array}{cc} E_{r}\; & \Sigma^{-1}L\\ 0\; & 0 \end{array}\right]U^{⁎}.$$

 □

Definition 3.1 Let $A,X\in C^{n\times n}$ and $\alpha_{A}=AA^{†}+{\left(A^{†}\right)}^{⁎}(E_{n}-AA^{†})$. If

(3.1) $$XAX=X,\;XA=A^{⋄}A,\;AX=AA^{⋄}\alpha_{A},$$

then the matrix *X* is called the strong B-T inverse of *A* and use the symbol $A_{s}^{⋄}$ to denote this inverse, where $A^{⋄}$ is the B-T inverse of *A*.

Theorem 3.2 *Let* $A\in C^{n\times n}$ *. Then, we have the strong B-T inverse* $A_{s}^{⋄}$ *of A is unique and* $A_{s}^{⋄}=A^{⋄}\alpha_{A}$ *.*

Proof Let $X_{1}$ and $X_{2}$ be two candidates of system (3.1), then

$$X_{1}=X_{1}AX_{1}=A^{⋄}AX_{1}=X_{2}AX_{1}=X_{2}AA^{⋄}\alpha_{A}=X_{2}AX_{2}=X_{2},$$

thus *X* is unique. Moreover,

$$X=XAX=A^{⋄}AX=A^{⋄}AA^{⋄}\alpha_{A}=A^{⋄}\alpha_{A}.$$

 □

Example 3.1 The strong B-T inverse $A_{s}^{⋄}$ is different from the MPWC inverse $A^{\circ }$*[7]*, CMP inverse $A^{c,†}$ *[9]**, MPCEP inverse*  *[3]**, Moore-Penrose inverse* $A^{†}$ *and MPBT inverse* $A^{†,⋄}$ *[11]**. CEPMP inverse*  *[6]* *and MPCEPMP inverse*  *[6]* Let $A=\left[\begin{array}{cccc} 1\; & 0\; & 0\; & 0\\ -i\; & 0\; & 0\; & 0\\ i\; & -i\; & 0\; & 0\\ i\; & 0\; & 0\; & 0 \end{array}\right]\in C^{4\times 4}$. Then it is easy to check that

$$\alpha_{A}=\left[\begin{array}{cccc} \frac{5}{9}+\frac{1}{9}i\; & \frac{2}{9}+\frac{2}{9}i\; & 0\; & \frac{1}{9}-\frac{2}{9}i\\ \frac{1}{9}-\frac{5}{9}i\; & \frac{2}{9}-\frac{2}{9}i\; & 0\; & -\frac{2}{9}-\frac{1}{9}i\\ \frac{1}{3}\; & -\frac{2}{3}i\; & 1\; & -\frac{1}{3}i\\ -\frac{1}{9}+\frac{5}{9}i\; & -\frac{2}{9}+\frac{2}{9}i\; & 0\; & \frac{2}{9}+\frac{1}{9}i \end{array}\right]$$

and

$$A_{s}^{⋄}=\left[\begin{array}{cccc} \frac{4}{15}\; & \frac{4}{15}i\; & -\frac{1}{5}-\frac{1}{5}i\; & -\frac{1}{15}i\\ -\frac{4}{15}i\; & \frac{4}{15}\; & -\frac{1}{5}+\frac{1}{5}i\; & -\frac{1}{15}\\ 0\; & 0\; & 0\; & 0\\ \frac{4}{15}i\; & -\frac{4}{15}\; & \frac{1}{5}-\frac{1}{5}i\; & \frac{1}{15} \end{array}\right],$$

however
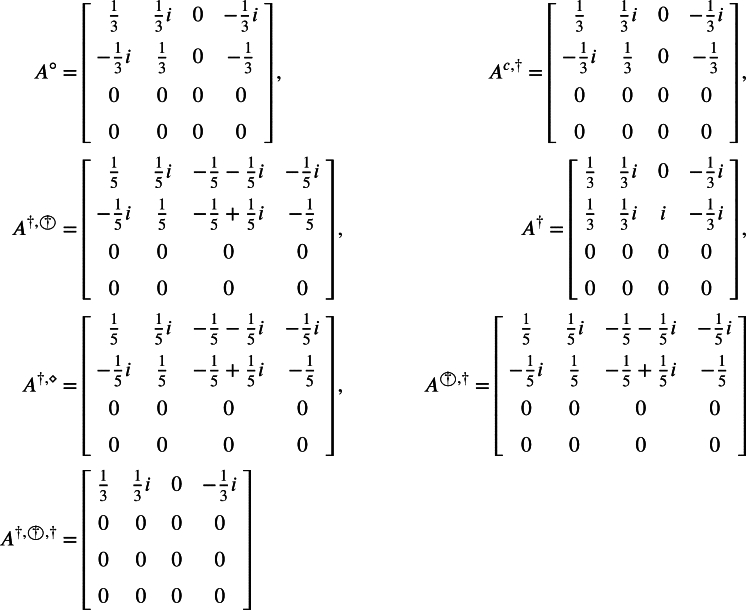

Theorem 3.3 *If* $A\in C^{n\times n}$ *has the decomposition as given in* (1.1) *, then the expression of the strong B-T inverse of A is*

(3.2) $$A_{s}^{⋄}=U\left[\begin{array}{cc} {\left(\Sigma K\right)}^{†}\; & {\left(\Sigma K\right)}^{†}\Sigma^{-1}L\\ 0\; & 0 \end{array}\right]U^{⁎}.$$

Proof By Lemma 3.1, we have

$$\alpha_{A}=U\left[\begin{array}{cc} E_{r}\; & \Sigma^{-1}L\\ 0\; & 0 \end{array}\right]U^{⁎},$$

where $E_{r}$ is the identity matrix of size *r*. By Theorem 3.2 and Lemma 2.2, we have

$$A_{s}^{⋄}=A^{⋄}\alpha_{A}=U\left[\begin{array}{cc} {\left(\Sigma K\right)}^{†}\; & 0\\ 0\; & 0 \end{array}\right]\left[\begin{array}{cc} E_{r}\; & \Sigma^{-1}L\\ 0\; & 0 \end{array}\right]U^{⁎}=U\left[\begin{array}{cc} {\left(\Sigma K\right)}^{†}\; & {\left(\Sigma K\right)}^{†}\Sigma^{-1}L\\ 0\; & 0 \end{array}\right]U^{⁎}.$$

 □

By Lemma 2.2 and equation (3.2), we have the B-T inverse of *A* coincides with the strong B-T inverse of *A* if and only if the condition $K^{⁎}L=0$ holds, which is the following Theorem 3.4.

Theorem 3.4 *If* $A\in C^{n\times n}$ *has the decomposition as given in* (1.1) *, then* $A^{⋄}=A_{s}^{⋄}$ *if and only if the condition* $K^{⁎}L=0$ *holds.*

Proof By Lemma 2.2 and equality (3.2), we have that the B-T inverse of *A* coincides with the strong B-T inverse of *A* if and only if

$${\left(\Sigma K\right)}^{†}\Sigma^{-1}L=0.$$

Moreover, ${\left(\Sigma K\right)}^{†}\Sigma^{-1}L=0\Leftrightarrow {\left(\Sigma K\right)}^{⁎}\Sigma^{-1}L=0\Leftrightarrow K^{⁎}L=0$. □

Several properties of the strong B-T inverse were given in the following proposition.

Proposition 3.5 *Let*$A,X\in C^{n\times n}$*. Then:*

**(1)** $A^{⋄}=A_{s}^{⋄}AA^{⋄}$*;*
**(2)** $A=\alpha_{A}A$*;*
**(3)** $A_{1}^{⋄}=AA_{s}^{⋄}A$*, where*$A_{1}^{⋄}=AA^{⋄}A$*;*
**(4)** $R(A_{s}^{⋄})=R(A^{⋄})$*;*
**(5)** $N(A_{s}^{⋄})=N(A^{⋄}\alpha_{A})$*;*
**(6)** $A\in A^{⋄}\alpha_{A}\{1\}$*.*

Proof (1). $A^{⋄}=A^{⋄}AA^{⋄}=A_{s}^{⋄}AA^{⋄}$ by the condition $A_{s}^{⋄}A=A^{⋄}A$ in Definition 3.1.

(2). $\alpha_{A}A=\left(AA^{†}+{\left(A^{†}\right)}^{⁎}(E_{n}-AA^{†})\right)A=A$.

(3). $A_{1}^{⋄}=AA^{⋄}A=AA_{s}^{⋄}A$ by the condition $A_{s}^{⋄}A=A^{⋄}A$ in Definition 3.1.

(4). $R(A^{⋄})\subseteq R(A_{s}^{⋄})$ by item (1) and $R(A_{s}^{⋄})\subseteq R(A^{⋄})$ is obvious by Theorem 3.2.

(5) is trivial by Theorem 3.2.

(6). $A^{⋄}\alpha_{A}AA^{⋄}\alpha_{A}=A^{⋄}AA^{⋄}\alpha_{A}=A^{⋄}\alpha_{A}$ by $A^{⋄}$ is an outer inverse of *A* and item (2). □

Several conditions were obtained in the following theorem, which answer the question when the outer inverse of *A* can be the strong B-T inverse of *A*.

Theorem 3.6 *Let* $A,X\in C^{n\times n}$ *. Then the following conditions are equivalent:*

**(1)** *X is the strong B-T inverse of A with* $X=A_{s}^{⋄}$ *;*
**(2)** XAX=X *,* $R(X)=R(A^{⋄})$ *and* $N(X)=N(A^{⋄}\alpha_{A})$ *;*
**(3)** XAX=X *,* $R(X)=R(AA^{†}A^{⁎})$ *and* $N(X)=N({\left(A^{⁎}\right)}^{2}\alpha_{A})$ *;*
**(4)** XAX=X *,* $R(X)\subseteq R(A^{⋄})$ *and* $N(X)\subseteq N(A^{⋄}\alpha_{A})$ *;*
**(5)** XAX=X *,* $R(X)\subseteq R(AA^{†}A^{⁎})$ *and* $N(X)\subseteq N({\left(A^{⁎}\right)}^{2}\alpha_{A})$ *.*

Proof (1)⇒(2) by Definition 3.1 and item (4) and (5) in Proposition 3.5.

(2)⇒(3). By Definition 1 in [2], one has $A^{⋄}={\left(A^{2}A^{†}\right)}^{†}$, which gives $R(A^{⋄})=R({\left(A^{2}A^{†}\right)}^{⁎})$ by p. 1910 in [4] and Proposition 6.1 in [4], that is $R(A^{⋄})=R(AA^{†}A^{⁎})$ by ${\left(AA^{†}\right)}^{⁎}=AA^{†}$. One has $N(X)=N(A^{⋄}\alpha_{A})$ is equivalent to $N(X)=N({\left(A^{2}A^{†}\right)}^{†}\alpha_{A})$ by $A^{⋄}={\left(A^{2}A^{†}\right)}^{†}$ in [2]. Moreover, $N({\left(A^{2}A^{†}\right)}^{†}\alpha_{A})=N({\left(A^{2}A^{†}\right)}^{⁎}\alpha_{A})=N(AA^{†}A^{⁎}\alpha_{A})=N({\left(A^{⁎}\right)}^{2}\alpha_{A})$. So, $N(X)=N({\left(A^{⁎}\right)}^{2}\alpha_{A})$.

(2)⇒(4) and (3)⇒(5) are obvious.

(4)⇒(1). The condition XAX=X implies $X(E_{n}-AX)=0$, so the condition $N(X)\subseteq N(A^{⋄}\alpha_{A})$ implies $A^{⋄}\alpha_{A}(E_{n}-AX)=0$, that is

(3.3) $$A^{⋄}\alpha_{A}=A^{⋄}\alpha_{A}AX=A^{⋄}AX.$$

$R(X)\subseteq R(A^{⋄})$ implies $X=A^{⋄}U$ for some $U\in C^{n\times n}$. So,

(3.4) $$X=A^{⋄}U=A^{⋄}AA^{⋄}U=A^{⋄}AX,$$

by $A^{⋄}AA^{⋄}=A^{⋄}$. Thus,

(3.5) $$X=A^{⋄}AX=A^{⋄}\alpha_{A},$$

by the equality (3.3) and (3.4). Therefore, *X* is the strong B-T inverse of *A* by (3.5) and Theorem 3.2.

(5)⇒(1). By the proof of (2)⇒(3), we have $R(A^{⋄})=R(AA^{†}A^{⁎})$ and $N(A^{⋄}\alpha_{A})=N({\left(A^{⁎}\right)}^{2}\alpha_{A})$. Thus, *X* is the strong B-T inverse of *A* by the proof of (4)⇒(1). □

Theorem 3.7 *Let* $A,X\in C^{n\times n}$ *. Then the following conditions are equivalent:*

**(1)** $X=A_{s}^{⋄}$ *;*
**(2)** $R(X)\subseteq R(A^{⋄})$ *and* $A^{⋄}AX=A^{⋄}\alpha_{A}$ *;*
**(3)** $N(A^{⋄}\alpha_{A})\subseteq N(X)$ *and* $A^{⋄}=XAA^{⋄}$ *.*

Proof (1)⇒(2). By Theorem 3.6, we have $R(X)\subseteq R(A^{⋄})$. In view of Theorem 3.2 and $A^{⋄}AA^{⋄}=A^{⋄}$, we have $A^{⋄}AX=A^{⋄}AA^{⋄}\alpha_{A}=A^{⋄}\alpha_{A}$.

(1)⇒(3). By Theorem 3.6, we have $N(A^{⋄}\alpha_{A})\subseteq N(X)$. $XAA^{⋄}=A^{⋄}\alpha_{A}AA^{⋄}=A^{⋄}AA^{⋄}=A^{⋄}$ by Theorem 3.2, Proposition 3.5 and $A^{⋄}AA^{⋄}=A^{⋄}$.

(2)⇒(1). We have $X=A^{⋄}U$ by $R(X)\subseteq R(A^{⋄})$, where $U\in C^{n\times n}$. Then

(3.6) $$X=A^{⋄}U=A^{⋄}AA^{⋄}U=A^{⋄}AX.$$

Thus,

$$X=A^{⋄}AX=A^{⋄}\alpha_{A}$$

by (3.6), which gives that $X=A_{s}^{⋄}$ by Theorem 3.2.

(3)⇒(1). We have $X=VA^{⋄}\alpha_{A}$ for some $V\in C^{n\times n}$ by $N(A^{⋄}\alpha_{A})\subseteq N(X)$. Then

(3.7) $$X=VA^{⋄}\alpha_{A}=VA^{⋄}\alpha_{A}AA^{⋄}\alpha_{A}=XAA^{⋄}\alpha_{A}$$

by item (6) in Proposition 3.5. Thus,

$$X=XAA^{⋄}\alpha_{A}=A^{⋄}\alpha_{A}$$

by the condition $A^{⋄}=XAA^{⋄}$ and equality (3.7), which gives that *X* is the strong B-T inverse of *A* by Theorem 3.2. □

Corollary 3.8 *Let* $A,X\in C^{n\times n}$ *. Then the following conditions are equivalent:*

**(1)** $X=A_{s}^{⋄}$ *;*
**(2)** $R(X)\subseteq R(A^{⋄})$ *and* $A^{⋄}\alpha_{A}AX=A^{⋄}\alpha_{A}$ *;*
**(3)** $R(X)\subseteq R(A^{⋄})$ *and* $A_{1}^{⋄}X=AA^{⋄}\alpha_{A}$ *;*
**(4)** $X=A^{⋄}AX$ *and* $A^{⋄}AX=A^{⋄}\alpha_{A}$ *;*
**(5)** $X=A^{⋄}AX$ *and* $A^{⋄}\alpha_{A}AX=A^{⋄}\alpha_{A}$ *;*
**(6)** $X=A^{⋄}AX$ *and* $A_{1}^{⋄}X=AA^{⋄}\alpha_{A}$ *;*
**(7)** $N(A^{⋄}\alpha_{A})\subseteq N(X)$ *and* $XA_{1}^{⋄}=A^{⋄}A$ *;*
**(8)** $X=XAA^{⋄}\alpha_{A}$ *and* $A^{⋄}=XAA^{⋄}$ *;*
**(9)** $X=XAA^{⋄}\alpha_{A}$ *and* $XA_{1}^{⋄}=A^{⋄}A$ *.*

Proof (1)⇔(2). The condition $A^{⋄}AX=A^{⋄}\alpha_{A}$ is equivalent to the condition $A^{⋄}\alpha_{A}AX=A^{⋄}\alpha_{A}$ by $A=\alpha_{A}A$ in Proposition 3.5. So (1)⇔(2) by Theorem 3.7.

(1)⇔(3). Pre-multiplying by *A* on $A^{⋄}AX=A^{⋄}\alpha_{A}$ gives $A_{1}^{⋄}X=AA^{⋄}\alpha_{A}$, Pre-multiplying by $A^{⋄}$ on $A_{1}^{⋄}X=AA^{⋄}\alpha_{A}$ gives $A^{⋄}AX=A^{⋄}\alpha_{A}$, which says that the condition $A^{⋄}AX=A^{⋄}\alpha_{A}$ if and only if $A_{1}^{⋄}X=AA^{⋄}\alpha_{A}$. So (1)⇔(3) by Theorem 3.7.

(1)⇔(4). $R(X)\subseteq R(A^{⋄})$ if and only if $X=A^{⋄}AX$. So (1)⇔(4) by Theorem 3.7.

(1)⇔(5). $R(X)\subseteq R(A^{⋄})$ if and only if $X=A^{⋄}AX$. So (1)⇔(5) can be proved by (1)⇔(2).

(1)⇔(6). $R(X)\subseteq R(A^{⋄})$ if and only if $X=A^{⋄}AX$. So (1)⇔(6) can be proved by (1)⇔(3).

(1)⇔(7). Post-multiplying by *A* on $A^{⋄}=XAA^{⋄}$ gives $XA_{1}^{⋄}=A^{⋄}A$, Post-multiplying by $A^{⋄}$ on $XA_{1}^{⋄}=A^{⋄}A$ gives $A^{⋄}=XAA^{⋄}$, which says that the condition $XA_{1}^{⋄}=A^{⋄}A$ is equivalent to $A^{⋄}=XAA^{⋄}$. So (1)⇔(7) by Theorem 3.7.

(1)⇔(8). The condition $N(A^{⋄}\alpha_{A})\subseteq N(X)$ is equivalent to $X=XAA^{⋄}\alpha_{A}$. So (1)⇔(8) by Theorem 3.7.

(1)⇔(9) can be got from the proof of (1)⇔(7). □

## Declaration of Competing Interest

The authors declare the following financial interests/personal relationships which may be considered as potential competing interests: Sanzhang Xu reports financial support was provided by 10.13039/501100009074Huaiyin Institute of Technology. Sanzhang XU reports a relationship with Huaiyin Institute of Technology that includes: employment and funding grants. If there are other authors, they declare that they have no known competing financial interests or personal relationships that could have appeared to influence the work reported in this paper.

## Data Availability

All relevant data are within the paper.
